# Secular trends in serum lipid profiles in young adults in Norway, 2001-19

**DOI:** 10.1016/j.athplu.2022.03.006

**Published:** 2022-03-30

**Authors:** Erik Kristoffer Arnesen, Kjetil Retterstøl

**Affiliations:** a Department of Nutrition, Institute of Basic Medical Sciences, University of Oslo, P.O.Box 1046 Blindern, 0317, Oslo, Norway; b The Lipid Clinic, Department of Endocrinology, Morbid Obesity and Preventive Medicine, Oslo University Hospital Aker, PO Box 4959, Nydalen, 0424, Oslo, Norway

**Keywords:** Cholesterol, Lipids, Dyslipidemia, Risk factors, Prevalence, Public health, TC, Total cholesterol, LDL-C, LDL cholesterol, HDL-C, HDL cholesterol, Non-HDL-C, non-HDL cholesterol, CHD, coronary heart disease, CVD, cardiovascular disease, LDL, Low-density lipoprotein, HDL, High-density lipoprotein

## Abstract

**Background:**

Lower prevalence of major cardiovascular disease (CVD) risk factors, such as dyslipidemia, hypertension and smoking, can explain a substantial part of the decline in CVD mortality and incidence for the past decades in Western countries. However, some studies have indicated less favorable trends in risk factors in recent years. We have assessed time trends in lipid profiles among young adults in Norway measured between 2001 and 2019.

**Methods:**

Samples of serum lipids analyzed at one large medical laboratory in Oslo, Norway, mainly requisitioned by primary care physicians, were analyzed cross-sectionally to estimate year-to-year trends among men and women aged 18–49 years. We also assessed the lipid distributions and proportions with adverse lipid levels.

**Results:**

In total, more than 2,6 million blood samples, comprising more than 1 million individuals (mean age 37.7 years) from all regions of Norway were included. All measures improved among all age groups in both women and men, especially in total and non-HDL cholesterol (-0.22 and -0.25 mmol/l per decade, respectively). There were downward shifts in the population distribution of total, non-HDL-C and LDL-C. The overall prevalences of total cholesterol ≥5.0 mmol/l and non-HDL-C ≥3.9 mmol/l similarly decreased, from ∼63 to 46% and from ∼52 to 34%, respectively. More than 1/3 had elevated levels of total and/or non-HDL-C in 2019.

**Conclusion:**

In a large proportion of the Norwegian population aged 18–49 years old, the lipid profiles improved during the last two decades. As the use of lipid-lowering medications is low in this age group, this likely reflects favorable secular trends.

## Highlights

• Adverse blood lipid profiles in young age is strongly associated with later atherosclerotic cardiovascular disease.
• Favorable trends in lipids have been observed in previous decades, but large-scale studies in young populations are lacking.
• Blood lipid profiles improved from 2001 through 2019 in 18–49-year-olds in Norway.
• The trends can likely not be attributed to use of lipid-lowering drugs.

## Introduction

Despite large reductions in mortality from atherosclerotic cardiovascular diseases (CVD) in Western countries for decades, it is still among the leading causes of death and population burden of disease [1]. A major part of the declining CVD mortality and incidence has been attributed to a lower prevalence of risk factors such as hypertension, dyslipidemia and smoking [2]. The Global Burden of Disease (GBD) study showed that the global exposure for high low density lipoprotein cholesterol (LDL-C) (i.e., above the theoretical minimum risk exposure level, 1.3 mmol/l) was 32.4% in 2019, more than for any other metabolic risk factor [3]. Concurrently, the NCD Risk Factor Collaboration estimated that high non-HDL-C (total cholesterol minus high-density lipoprotein (HDL) cholesterol) caused 3.9 million deaths from ischaemic heart disease (IHD) and ischaemic stroke in 2017 [4].

During the past four decades Norway has turned from a very high CVD risk country to a low risk country [5]. A lower prevalence of high total cholesterol (TC) or high TC/HDL-C ratio has been proposed to account for much of the decline in both the incidence and mortality of coronary heart disease (CHD) and sudden deaths from CHD in Norway for the latest decades [6,7] and in other countries. This reduction in cholesterol levels has partly been credited to lipid-lowering medications, but environmental/life style factors may also have played an important role, as the reduction started well before lipid-lowering medications became common, and also occurred among non-users [8]. As the decline in myocardial infarction rates has occurred in practically all groups, both young and old, it likely reflects effects of primary or primordial preventive approaches. Dietary changes, especially a shift from saturated fatty acids (SFA) and *trans*-fatty acids (TFA) to unsaturated fats, likely contributed to the reduction in serum cholesterol before the “statin era” [4,9]. The percentage of energy intake from total fats and SFA in the Norwegian diet was reduced from the 1970's up to the 1990's, like in many other western countries. The current consumption of SFA is still higher than recommended, while the intake of polyunsaturated fats (PUFA) is lower, partly caused by a regular increase in the consumption of cheese, cream and meat products. Whether this is reflected in the trends in cholesterol levels in the population is unknown.

Recently, international studies have suggested that CVD mortality rates have flattened out among young and middle-aged persons since year 2000, or even increased in some cases, which has been explained by unfavorable risk factor trends [10]. Declines in both IHD incidence and mortality in Western-European countries appeared to become less steep after 2009 in all geographical regions [11]. On the contrary, the incidence of acute myocardial infarction declined by 1.7% per year in young adults (25–44 years of age) in Norway, with a steeper decline from 2009 (5.3% per year) [12].

To explain this development, it is necessary to assess the prevalence of risk factors over time. However, nationwide data for cholesterol is not available in Norway. Published surveys from recent years are generally limited to certain geographic areas or single cross-sectional studies that cannot demonstrate time trends [7,13,14]. Such health surveys include self-selected participants who may have higher education levels and a healthier lifestyle than non-participants, obscuring important group differences in risk factors. Trends in LDL-C or non-HDL-C have rarely been reported. Non-HDL-C is strongly associated with long-term risk of CVD [15], and has been shown to yield a more correct risk classification than both calculated and directly measured LDL-C. As non-HDL-C encompass both LDL-C, VLDL-C, IDL-C and remnant cholesterol, it is also a more convenient measure of atherogenic lipoproteins than LDL-C alone, which is usually calculated by the Friedewald equation [16]. Non-HDL-C can be assessed non-fasted and does not require triglyceride (TG) levels below 4.5 mmol/l, as in the Friedewald equation.

In the present study we estimated the 20-year trends in serum lipids among young Norwegian adults (18–49-year-olds) using clinical laboratory data as the data source. We also assessed shifts in the population distribution of lipid levels. To minimize the likely effect of increased prescription of lipid-lowering medication, we restricted our population to younger than 50 years of age. Another reason to examine adults below 50 years of age is the strong association between cholesterol levels in young age and risk of CVD several decades later [17,18]. Consequently, early detection of elevated cholesterol or dyslipidemia is important.

## Methods

We retrospectively assessed population trends in serum lipids from 2001 to 2019 among young Norwegian adults (aged 18 through 49 years) using deidentified test results extracted from a large clinical laboratory as the data source.

The population includes adults with valid serum lipid data measured from 2001 throughout 2019. To minimize the likely effect of increased prescription of lipid-lowering medications, we restricted our population to below 50 years of age, in which the use is typically very low (<5%) (See Supplementary Fig. 1). The number of tests per individual was restricted to maximum 2 per year (less than 1% had more than 4 tests per year), and subjects with more than one test on the same date were excluded.

Blood samples in our study were mainly requisitioned by general practice or occupational health service physicians; all residents in Norway are entitled to a general practitioner through the National Insurance scheme.

### Data collection

The data were retrieved from the laboratory database from *Fürst Medisinsk Laboratorium,* Oslo, Norway, which is the largest medical laboratory in Norway and of laboratories has the largest coverage in Norway, performing about 26 million analyzes in 2019. The main laboratory is located in Oslo, Norway. Test samples are sent from all over the country, but the majority are from Oslo and surrounding areas in the South-Eastern part of Norway.

The database included the patients' personal identification number (de-identified in the extracted file) and age, testing date, test results and postal code of the medical center, which was used as a proxy for the patients’ place of residence. All available laboratory results for the variables of interest included in the database between February 2001 and December 31st^,^ 2019 were extracted for analysis.

### Ethics

All patient data were deidentified before they were handed from the laboratory to the University of Oslo's Services for Sensitive Data (TSD). Employees at the medical laboratory had access to the laboratory data system according to rights and duties in the health personnel act. A data protection impact assessment (DPIA) was performed and approved by the internal privacy protection deputy at the University of Oslo in compliance to the EU General Data Protection Regulation. The project was approved by the Norwegian Regional Committee for Medical and Health Research Ethics (ref. 2016/1693). No patients were involved in the study design or analysis.

### Laboratory analyses

All laboratory analyzes were performed centralized using one type of methodology at Fürst's central laboratory in Oslo. The same analytic methods have been used over long periods, and conversion to new methods have been validated through continuous quality monitoring, as performed by mandatory external quality assessments. The laboratory is accredited according to ISO 15189:2012 requirements for quality and competence in medical laboratories, and is subjected to several external quality controls each year and daily internal quality controls.

Measures included for this study were serum total-, HDL- and LDL-C and TG. We had no information on whether samples were drawn were in the fasted state. However, during this time period, 8h fasting was recommended.

For consistency, only calculated LDL-C with Fridewald equation, excluding patients with TG ≥ 4.5 mmol/l and calculated LDL-C ≤1.8 mmol/l is reported, as direct LDL-C measures were not available for the first years. However, it was a very high correlation between directly measured and calculated LDL-C. Serum lipids were measured with enzymatic colorimetric methods on automated instruments from Roche (Modular P) until September 2009 and then from Siemens (Advia Chemistry). Standardization, calibration and updated versions/generations were used as recommended by the manufacturers. The analytic coefficient of variation from daily internal quality controls were typically ≤2%. External quality assessment was performed ≥4 times each year, demonstrating quality within the accept-range throughout the period (Labquality Oy, Finland).

We further categorized lipid values as non-optimal (i.e., elevated) using cut-offs from the most recent guidelines [19]: TG ≥ 1.7 mmol/l, TC ≥ 5 mmol/l, non-HDL-C ≥3.9 mmol/l, and LDL-C ≥3 mmol/l. HDL-C was categorized as low if ≤ 1 mmol/l in men and ≤1.2 mmol/l in women. The proportion of tests with TC > 7.0 mmol/l and/or LDL-C >5.0 mmol/l was also calculated; these indicate eligibility for statin treatment regardless of total CVD risk score [20].

### Statistical analyzes

Demographical characteristics are presented as frequencies and percentages, means with standard deviations or medians with 25th-75th percentiles. Data are shown combined and separately for women and men and age categories (18–19, 20–29, 30–39, and 40–49 years).

Differences in means of lipid values between years were examined by analyses of variance (ANOVA) for continuous variables, and χ^2^ test for categorical variables. To estimate time trends in each of the lipid variables, mixed-effects regression models stratified by sex, age group and region were used (alpha <0.01), with calendar year as predictor and person ID as a random intercept to account for intraindividual correlations. Similarly, mixed-effets logistic regression was used for categorical endpoints. An interaction term between sex and year was also included in the models to test for significant differences between women and men, age groups or regions. Continuous outcome variables are presented as means with 95% CI or medians with 25th–75th percentiles. TG values were log transformed before the regression analysis. The 10th, 50th and 90th percentiles in lipid variables were calculated to estimate changes in the population distributions, visualized by Epanechnikov kernel density distribution curves. All analyses were performed using Stata software, version 17 (Stata Corporation, Texas, USA).

## Results

The final sample consisted of 1,010,658 individuals (∼50% women) and 2,639,907 tests taken during the whole period. The number of eligible tests increased yearly, from 58,070 in 2001 to 192,608 in 2019. Most tests were taken at physicians' offices, while 6.4% were taken at the laboratory's facilities. There were in total 2,580,059 measurements of TC, 2,231,085 of HDL-C, and 976,014 of TG from the patients in the included sample. Calculation of non-HDL-C was possible from 2,206,893 measurements.

The mean age of the patients was 37.7 years (±8,5), median 39 years. Only 2% were below 20 years of age. As shown in Table 1, almost half of the population was 40 years old or higher. The mean age and age distribution was stable during the period, but the proportion of patients between 18 and 29 years increased. As expected, a large majority of tests, 84%, were from the South-East of Norway, which also has the largest population size.

**Table 1:** Demographic characteristics of the sample analyzed in 2001 and 2019.

	2001	2019
N	53,664	171,389
% women	49.0	51.7
Age, mean (SD)	38.0 (8.0)	37.3 (8.7)
***Age groups (%)***		
<20	1.3	2.5
20-29	17.9	21.5
30-39	35.1	31.5
40-49	45.8	44.5
***Region (%)***		
East	85.2	82.8
South	4.7	2.0
West	8.4	7.5
Mid	0.2	7.0
North	1.5	0.8

### Lipid levels in 2019 vs. 2001

**Fig. 1:**
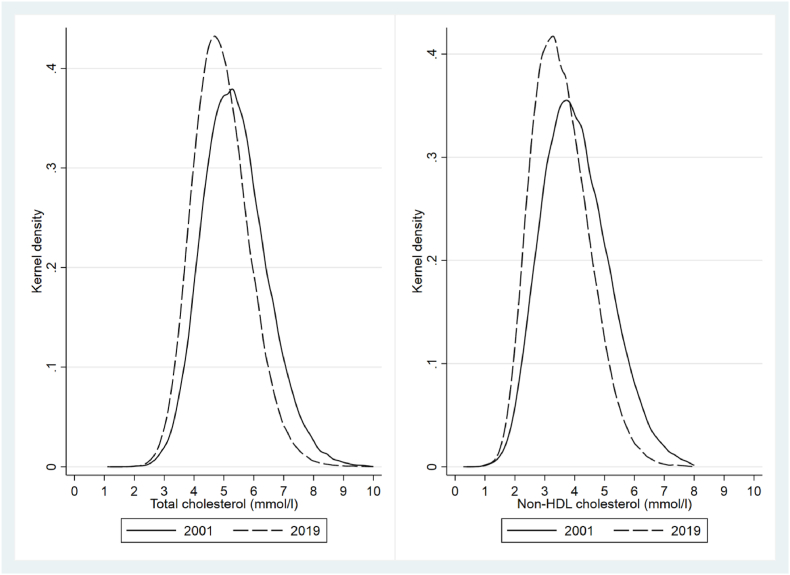
Population distributions of serum total (left) and non-HDL cholesterol (right) in 18–49 year old men and women in 2001 (solid lines) and 2019 (dashed lines).

There were test results from 53,666 individuals in 2001 and from 171,419 in 2019. The sex-specific means and distributions of the lipid parameters in 2001 and 2019 are shown in Table 2 and Fig. 1 (distribution curves for LDL-C, HDL-C and TG are shown in Supplementary Fig. 2). There was a shift towards significantly lower values in the whole distribution of TC and non-HDL-C in men and women and in all age groups.

**Table 2:** Lipid profiles in 18–49 year old men and women in Norway in 2001 and 2019, and mean/median changes (with 95% confidence intervals) between 2001 and 2019.

	Women					Men				
**2001**										
	N tests	Mean (SD)	Median	25-%	75-%	N tests	Mean (SD)	Median	25-%	75-%
Total cholesterol, mmol/l	28,206	5.27 (1.03)	5.20	4.55	5.90	29,020	5.49 (1.12)	5.40	4.70	6.20
HDL-C, mmol/l	21,499	1.52 (0.38)	1.49	1.25	1.76	22,625	1.24 (0.31)	1.20	1.03	1.40
Non-HDL-C, mmol/l	21,375	3.80 (1.08)	3.68	3.03	4.44	22,484	4.30 (1.15)	4.24	3.49	5.05
LDL-C, mmol/l	9538	3.30 (0.98)	3.20	2.60	3.88	10,231	3.56 (1.03)	3.50	2.84	4.21
Triglycerides, mmol/l	11,035	1.36 (1.00)	1.12	0.81	1.61	11,813	1.95 (1.56)	1.58	1.09	2.31
**2019**										
	N tests	Mean (SD)	Median	25-%	75-%	N tests	Mean (SD)	Median	25-%	75-%
Total cholesterol, mmol/l	98,271	4.84 (0.89)	4.80	4.20	5.40	90,660	5.00 (1.01)	4.95	4.30	5.60
HDL-C, mmol/l	87,893	1.54 (0.39)	1.49	1.25	1.77	82,039	1.24 (0.31)	1.20	1.03	1.41
Non-HDL-C, mmol/l	87,193	3.32 (0.87)	3.22	2.69	3.85	81,491	3.78 (1.00)	3.72	3.06	4.43
LDL-C, mmol/l	22,869	2.87 (0.74)	2.77	2.32	3.32	21,865	3.10 (0.84)	3.03	2.49	3.63
Triglycerides, mmol/l	28,241	1.25 (0.84)	1.05	0.77	1.48	27,286	1.82 (1.65)	1.42	0.98	2.14
**Change from 2001 to 2019, mean (95% CI)**										
	**Women**					**Men**				
Total cholesterol	-0.427 (-0.414, -0.439)					-0.485 (-0.471, -0.499)				
HDL-C	0.015 (0.009, 0.021)					0.007 (0.003, 0.012)				
Non-HDL-C	-0.481 (-0.468, -0.495)					-0.529 (-0.513, -0.544)				
LDL-C	-0.439 (-0.420, -0.458)					-0.464 (-0.443, -0.485)				
Triglycerides (median)	-0.070 (-0.086, -0.054)					-0.160 (-0.136, -0.184)

TC, non-HDL-C, LDL-C and TG increased significantly with age. Men had higher levels of TC, non-HDL-C, LDL-C and TG, while women had higher levels of HDL-C. However, in the <20 years age group, women had higher TC and LDL-C than men.

Table 3 shows the mean differences between 2001 and 2019 by sex, 10-year age group and region. The crude mean TC and non-HDL-C levels decreased between 2001 and 2019, from 5.38 (±1.08) to 4.92 (±0.95) mmol/l and from 4.06 (±1.15) to 3.54 (±0.96) mmol/l, respectively. Comparing the first five years (2001–05) to the last five years (2015–19), age-adjusted mean TC decreased by 0.25 mmol/l (95% CI 0.25, 0.25) and non-HDL-C decreased by 0.28 mmol/l (95% CI 0.27, 0.28).

**Table 3:** Age- and sex-adjusted changes in mean cholesterol and triglycerides (mmol/l) (with 95% confidence intervals) from 2001 through 2019.

		Total cholesterol	HDL-C	Non-HDL-C	LDL-C	Triglycerides
All		-0.472 (-0.464, -0.479)	0.042 (0.039, 0.044)	-0.545 (-0.537, -0.553)	-0.491 (-0.479, -0.504)	-0.142 (-0.124, -0.161)
Women		-0.380 (-0.370, -0.389)_a_	0.041 (0.037, 0.045)_a_	-0.442 (-0.432, -0.453)_a_	-0.428 (-0.411, -0.444)_a_	-0.078 (-0.096, -0.061)_a_
Men		-0.555 (-0.544, -0.567)_a_	0.026 (0.022, 0.029)_a_	-0.617 (-0.605, -629)_a_	-0.536 (-0.518, -0.555)_a_	-0.177 (-0.144, -0.209)_a_
***By age group (sex-adjusted)***						
<20 y	-0.242 (-0.178, -0.307)		0.008 (-0.021, 0.037)	-0.279 (-0.202, -0.356)	-0.202 (-0.086, -0.317)	-0.089 (-0.200, 0.022)
20-29 y	-0.218 (-0.200, -0.235)		0.043 (0.036, 0.051)_b_	-0.292 (-0.272, -0.313)	-0.234 (-0.202, -0.265)	-0.174 (-0.135, -0.212)
30-39 y	-0.284 (-0.271, -0.297)_b_		0.032 (0.028, 0.037)_b_	-0.337 (-0.322, -0.352)	-0.381 (-0.358, -0.403)_b_	-0.091 (-0.056, -0.125)
40-49 y	-0.395 (-0.383, -0.407)_b_		0.063 (0.059, 0.067)_b_	-0.455 (-0.442, -0.468)_b_	-0.492 (-0.473, -0.510)_b_	-0.050 (-0.021, -0.078)
***By region (age + sex adjusted)***						
East	-0.458 (-0.450, -0.466)		0.037 (0.034, 0.040)	-0.529 (-0.520, -0.538)	-0.477 (-0.464, -0.490)	-0.137 (-0.117, -0.157)
South	-0.467 (-0.424, -0.510)		0.015 (0.001, 0.029)	-0.488 (-0.440, -0.535)	-0.381 (-0.305, -0.457)_c_	-0.014 (-0.123, 0.095)
West	-0.569 (-0.541, -0.597)_c_		0.017 (0.008, 0.027)_c_	-0.614 (-0.582, -0.646)_c_	-0.653 (-0.603, -0.703)_c_	-0.180 (-0.110, -0.251)
Mid	-0.694 (-0.522, -0.866)_c_		0.014 (-0.037, 0.065)	-0.671 (-0.497, -0.845)	-0.644 (-0.434, -0.853)	-0.303 (-0.665, 0.059)
North	-0.496 (-0.423, -0.568)		-0.059 (-0.038, -0.081)_c_	-0.455 (-0.377, -0.532)	-0.451 (-0.309, -0.593)	0.034 (-0.172, 0.240)

a Significant difference between men and women (*p* < 0.01).

b Significant difference in change compared to age group <20 years (*p* < 0.01).

c Significant difference in change compared to Eastern region (*p* < 0.01).

### Time trends from 2001 through 2019

**Fig. 2:**
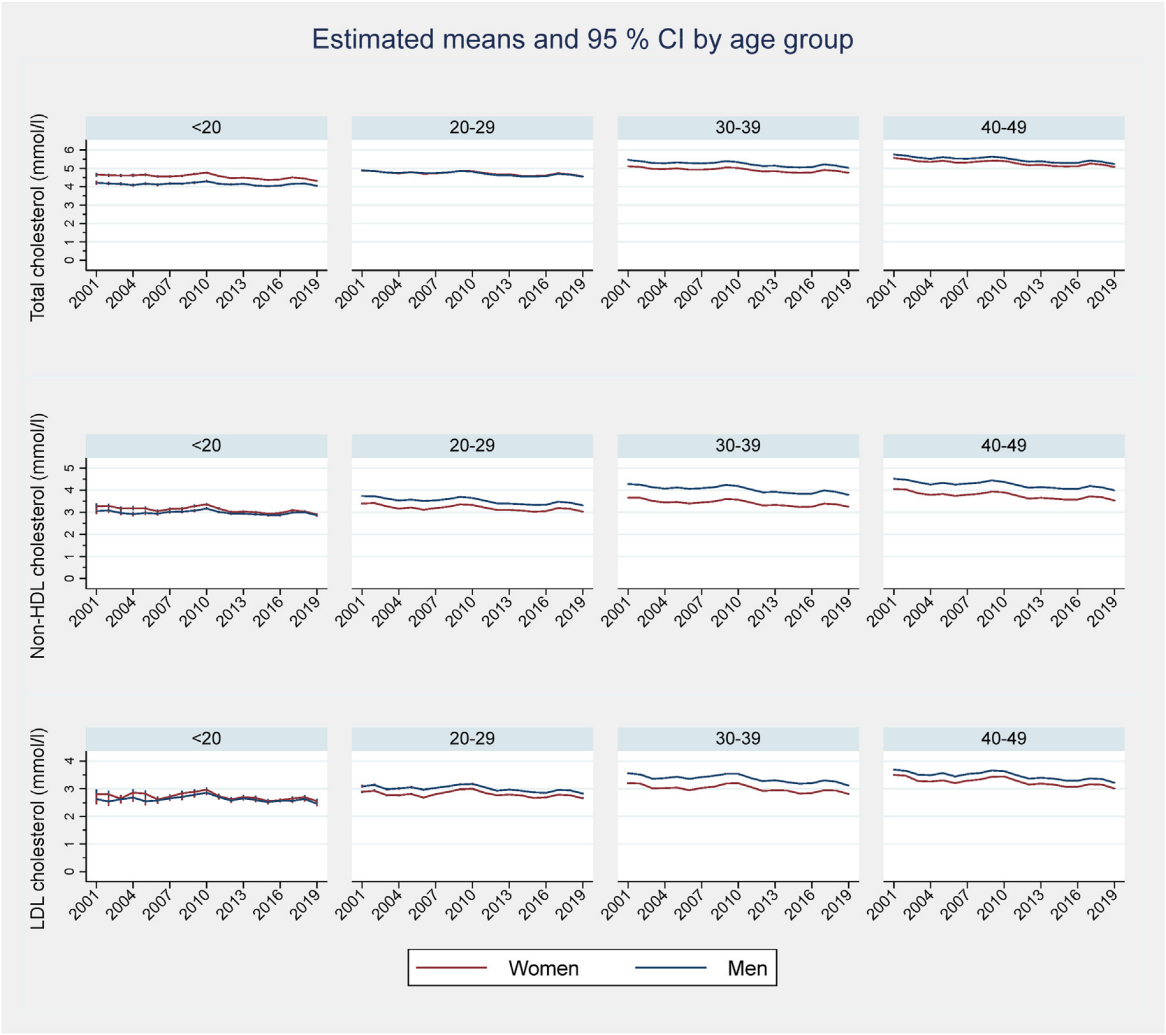
Trends in mean total cholesterol (top), non-HDL cholesterol (middle) and LDL cholesterol (bottom) levels in 18–49 year olds by sex and age group, 2001 through 2019. 95% confidence intervals are shown as spikes.

Fig. 2 shows the yearly trends in TC, non-HDL-C and LDL-C from 2001 to 2019 by sex and age group (trends in HDL-C and TG are shown in Supplementary Fig. 3).

The time trends were favorable and changed significantly across age groups and geographic regions, in both men and women. However, the trends in HDL-C (Supplementary Fig. 3) were very small in absolute terms, and not significant in the <20 years age group. The decrease in mean non-HDL-C was similar to calculated LDL-C in direction and magnitude.

The mean age-adjusted reduction in TC was 0.216 mmol/l (95% CI -0.214, -0.219) per decade (*p* < 0,0001). This reduction was slightly lager in men (-0.266 mmol/l (95% CI -0.262, -0.269)) than in women (-0.163 mmol/l (95% CI -0.160, -0.166)). The year-to-year age-adjusted reductions in TC and non-HDL-C diminished with time, but were still significant (e.g., TC -0.073 mmol/l (95% CI -0.070, -0.076) per year from 2001 to 2004 and -0.010 mmol/l (95% CI -0.010, -0.011) per year from 2013 to 2019). For mean non-HDL-C, the age-adjusted change per decade was -0.191 (95% CI -0.188, -0.194) mmol/l in women and -0.301 (95% CI -0.297, -0.304) mmol/l in men. TG also decreased, and HDL-C increased, in the age-adjusted analyses, but the changes were negligible: -0.044 (95% CI -0.38, -0.049) and +0.026 mmol/l (95% CI 0.025, 0.026) per decade, respectively.

### Prevalence of adverse lipid levels

The sex- and age-specific trends in the prevalence of elevated TC, non-HDL-C, LDL-C and TG, and low HDL-C, are shown in Supplementary Table 1. Overall and in all subgroups, the prevalence decreased linearly. The odds for TC levels ≥5.0 mmol/l was 0.53 (95% CI 0.51, 0.55) in 2019 compared to 2001. Men were more likely than women to have TC levels ≥5.0 mmol/l. There was also a significant decrease in the prevalence of TC ≥ 7.0 mmol/l (from 8.1% in 2001 to 2.8% in 2019), and in the prevalence of LDL-C ≥5 (from 7.1 to 1.5%) (not shown).

While the overall prevalence of TC ≥ 5 mmol/l decreased to below 50%, the age-adjusted LDL-C in 2019 remained high (i.e. ≥3 mmol/l) in 43% (37% in women, 49.5% in men). On the other hand, the age-adjusted prevalence of non-HDL-C ≥3,9 mmol/l decreased to 31.2% (95% CI 31.0, 31.4) in 2019.

## Discussion

This study of more than 2,6 million blood samples from 18 to 49-year-olds indicates that the decline in serum total cholesterol in Norway has continued through the two latest decades. The decline was mostly driven by non-HDL-C and occurred across the whole population distribution.

Our findings are consistent with longer international trends that has shown decreasing TC levels, with the largest decreases occurring in northwestern Europe [4]. Although the global age-standardized non-HDL cholesterol levels were almost unchanged from 1980 to 2018, they decreased by > 0.3 mmol/l per decade in northwestern Europe [4]. The slightly lower decrease in our study likely reflects the different age and time span, and that the non-HDL cholesterol levels had already decreased substantially before 2001. Regarding the Norwegian population, the Tromsø study from Northern Norway found a 14% decrease in mean TC from 1994 to 2008, and a 42% decrease in the prevalence of high total/HDL-C ratio [7]. Likewise, the prevalence of elevated TC decreased in the central part of Norway, but 40–50% in the age group 20–39 years had TC above 5 mmol/l in 2006–08, quite similar to our findings [13].

On the other hand, we could not confirm findings from some population-based surveys that indicated increasing trends or a plateau in cholesterol levels. In neighboring countries, age-adjusted mean TC levels in one region in Sweden decreased between 1990–95 and 2002–07, but then increased among both women and men in rural and urban areas [21]. The prevalence of hypercholesterolaemia also increased from 2002–07 to 2008–10, but trends in LDL nor HDL-C were not reported. While TC levels in some areas in Finland decreased as much as 20–23%, they seemed to increase after 2007, especially among women [22]. This occurred in parallel with increasing consumption of butter-fat, while the use of skimmed milk declined, and the use of lipid-lowering medications was stable. Another Finnish study of a younger population found that previously observed beneficial trends in LDL-C leveled off after 2007 [23].

### Potential mechanisms

As the use of lipid-lowering medication is very limited in the young age groups we have studied (see Supplementary Fig. 1), we are tempted to speculate that these trends mostly represent extensive and favorable behavioral and environmental changes. This could be related to cohort or period effects, or both, which we have not estimated. As shown in the distribution plots, the decline in TC and non-HDL-C occurred across the entire range, suggesting population-wide influences [24].

Some potentially contributing dietary changes could be improved dietary fat quality, increased intake of dietary fibre, and reduced consumption of unfiltered coffee. According to household survey data and wholesale statistics for Norway, there were relatively small changes in the intake of SFA and PUFA during this time period, SFA varying between 14 and 15% of total energy intake (E%). Each 1 E% replacement of SFA with PUFA is predicted to lower TC and LDL-C by ∼0.06 mmol/l [25]. The consumption of industrially produced TFA has been declining for 25 years, and a regulation to limit the content of TFA in foodstuff was implemented in Norway in 2014. There are few individual-level dietary data on trends in these nutrients, so these ecological associations cannot be causally interpreted. Increased popularity in high-fat, low-carbohydrate diets has raised concern because of the potentially adverse effects on blood lipids of a high SFA intake [26,27]. From the present data, these effects do not seem to have manifested on the population scale, but may have been detected in specific strata. A substantial decrease in the prevalence of smoking may also have contributed to the favorable changes [28].

Some of the trends may even seem paradoxical considering the increased prevalence of overweight and obesity in the population [29], which would be expected to increase TG and lower HDL-C-levels whereas we observed only very small changes. National data on overweight/obesity trends in young adults in Norway are, however, sparse (Supplementary Fig. 4), but similar trends in TG and HDL-C have been reported in other similar populations [4,23]. Both TG and HDL-C is affected by several lifestyle factors, including diet. The average dietary consumption of added sugars has decreased considerably, which may explain some of the beneficial trends in TG and HDL-C. Further, the consumption of industrial TFA, which are inversely associated with HDL-C, has been practically eliminated. Again, the lower prevalence of smoking may also be an attributable factor.

### Strenghts and limitations

Methodological period effects also warrant consideration. The use of similar analytical methods over time, subject to internal and external quality controls, should lessen concerns that the trends reflect a purely methodological artifact. While there was a change in the instruments used in 2009, we could not see any sudden drop or rise in the lipid values related to this. Further, the observed trends are in line with findings from other settings with comparable age groups [13,14,30].

The principal limitations of this study are the non-random sampling of the population and that the reasons for the individuals’ tests is unknown. As the indications for assessing blood lipids are many, our sample does not necessarily represent a homogenous group with an unusually adverse lipid profile. If so, this bias should be constant over time, but requisition practices may have changed during the period. Therefore, we do not claim that the absolute lipid levels in our population is representative for all Norwegians 18–49 years old. We also had no possibility to control for potential pre-analytic sources of errors, such as variation during the day, weight loss, pregnancy, medications or any chronic or acute disease. Again, this is not highly relevant in trend analyses from the same laboratory. We could not verify whether the tests were of fasting or non-fasting patients, but non-fasting lipid profiles are now recommended by international guidelines for TC, HDL-C and LDL-C (alternatively, non-HDL-C), and the differences in TG are likely too small to be meaningful on a population level [31].

The large sample size also makes the estimates robust to outliers and random errors. It has previously been demonstrated that the use of large data sets from clinical laboratories can be valuable for epidemiologic research and yield similar distributions of lipid values to population-based surveys [32,33].

We report LDL-C levels calculated by the Friedewald equation [16]. Even though this method is known to underestimate the reference method and directly measured LDL-C, this is unlikely to affect the trends, which is of interest in this study. The underestimation has been shown to increase not only with higher TG, but also with lower LDL-C levels, therefore we excluded subjects who had direct measures of LDL-C ≤1,8 mmol/l when calculating LDL-C, as recommended by the European Atherosclerosis Society [34]. Furthermore, the trends in calculated LDL-C corresponded to the trends in non-HDL-C in our sample, which may be a better predictor of CVD [34].

### Implications

An important contribution of this study is the inclusion of young adults, whom are typically not candidates for blood lipid assessments even though there is a strong association between cholesterol levels in young age and risk of CVD [17]. CVD is associated with an accumulated risk factor exposure, and high (LDL) cholesterol levels in early life is more strongly associated with CVD risk than cholesterol measured at later ages [18,35]. In Norway there is no routine lipid measuring in people below 40 years of age. Treatment of high lipid levels is not recommended based on the lipid profile alone, but on the total risk score, which is generally low in young patients. However, not only low cholesterol, but also the cholesterol burden through the life-course is important, even when the calculated 10-year risk is low [36].

Meta-analyses of prospective cohort studies have found that each 1 mmol/l increase in TC is associated with relative risks (RR) of 1.7 for stroke and 2.2 for ischaemic heart disease (IHD) in 35–44 year-olds [37]. Lifelong exposure to lower LDL-C levels are associated with near to 50% lower risk of CHD per 1 mmol/l (33% lower risk per 0.5 mmol/l) in Mendelian randomized studies, i.e., almost 3-fold higher effects than the same reduction achieved through statin treatment later in life [38,39], supporting the concept of “the longer the better” regarding atherogenic lipid lowering [40]. From this it can be assumed that a 0.5 mmol/l reduction of LDL-C, from a baseline of 2 or 3 mmol/l, will lower the 20-year risk of CHD by 21% over 20 years, while a 1 mmol/l reduction would alone lower the risk by 38% [39].

In one recent Norwegian population-based cohort, the age- and sex-adjusted incidence of first CHD decreased by 51% (3% per year) between 1995 and 2010, while CHD mortality declined by ∼7% annually [7]. A 14% decrease in TC during the same period accounted for 32% of the decreased incidence in that population. In Sweden, lower TC levels (mean 0.64 mmol/l, or 10%) in the population explained 53.7% of the decline in CHD mortality between 1986 and 2002 in primary prevention, the TC cholesterol reduction being largely due to dietary changes [41].

## Conclusion

In the largest study of long-term trends in blood lipid levels in any Norwegian population to date, we found in this sample of young and middle-aged adults a generally favorable trend between 2001 and 2019 in TC, non-HDL-cholesterol and LDL-cholesterol, and no unfavorable trend in HDL-C or TG. In 2019, the mean TC and non-HDL-C were within the recommended thresholds, although the prevalence of elevated lipids was still around 50% in the 40-49-year-old group. This may indicate reasons to reinforce population-targeted preventive efforts to improve risk factors, including dietary interventions. The use of lipid-lowering medications is low in these age groups, suggesting a major contribution from other mass population-wide changes, despite the worrying simultaneous increase in overweight and obesity.

## Author contributions

EKA and KR designed the hypotheses and study framework; EKA obtained funding, collected the data and conducted the analyses, and wrote the manuscript; KR contributed with interpretation of the data and revision of the manuscript.

## Financial support

The project has been made possible by funding from the LHL Heart Fund (LHLs Hjertefond). The sponsors were not involved in study design, data collection or any other parts of the study.

## Declaration of competing interest

The authors declare the following financial interests/personal relationships which may be considered as potential competing interests:

KR has received research grants and/or personal fees from Akcea, Amgen, Sanofi and Sunnovion, none of which are related to the content of this manuscript.
