# Supplementary material for: Secular trends in serum lipid profiles in young adults in Norway, 2001-19
Source: Atheroscler Plus. 2022 Mar 30;48:60–7. doi: 10.1016/j.athplu.2022.03.006 (PMC9833235; doi:10.1016/j.athplu.2022.03.006)
Supplement: Multimedia component 1 [file mmc1.docx]

SUPPLEMENTARY MATERIAL

**Table S1.** Age and sex adjusted prevalence of elevated total, non-HDL and LDL cholesterol and triglycerides, and low HDL cholesterol, in 2001 and 2019_a_.

|  | TC  ≥5.0 mmol/l | | | | | Non-HDL-C ≥3.9 mmol/l | | | | | LDL-C ≥3.0 mmol/l | | | | HDL-C <1.3 mmol/l (men) | | | HDL-C <1.0 mmol/l (women) | | | | | | TG >1.7 mmol/l | |
| --- | --- | --- | --- | --- | --- | --- | --- | --- | --- | --- | --- | --- | --- | --- | --- | --- | --- | --- | --- | --- | --- | --- | --- | --- | --- |
|  | 2001 | | 2019 | | | 2001 | | | 2019 | | 2001 | | 2019 | | 2001 | | 2019 | 2001 | | | 2019 | | | 2001 | 2019 |
| *Age-adjusted* | | | | | | | | | | | | | | | | | | | | | | | | | |
| All (%) | 62.7 | | | 44.8 | | 51.0 | | 31.2 | | | | 64.4 | | 43.1 |  | | | | | | | | | 32.5 | 26.1 |
| Women (%) | 57.6 | | | 41.5 | | 39.0 | | 22.2 | | | | 57.6 | | 37.0 |  | | | | 6.6 | 5.2 | | | | 20.2 | 16.9 |
| Men (%) | 67.7 | | | 48.2 | | 62.4 | | 40.5 | | | | 70.8 | | 49.5 | 64.6 | | 62.4 | |  | | | | | 44.3 | 35.8 |
| ***By age group*** *(sex-adjusted)* | | | | | | | | | | | | | | | | | | | | | | | | | |
| <20 y (%) | 25.3 | 15.4 | | | 19.5 | | 9.1 | | | 31.0 | | | 19.0 | | | 53.5 | 52.9 | | 13.3 | | | 15.0 | 16.3 | | 12.1 |
| 20-29 (%) | 41.1 | 29.4 | | | 30.0 | | 18.1 | | | 42.0 | | | 30.3 | | | 46.4 | 43.6 | | 13.3 | | | 11.9 | 23.5 | | 17.5 |
| 30-39 (%) | 56.6 | 44.4 | | | 44.9 | | 31.5 | | | 60.0 | | | 43.1 | | | 49.7 | 46.8 | | 15.4 | | | 13.6 | 29.9 | | 26.5 |
| 40-49 (%) | 72.4 | 58.0 | | | 58.6 | | 42.4 | | | 71.5 | | | 51.9 | | | 46.6 | 44.7 | | 14.0 | | | 12.9 | 35.2 | | 32.4 |

_a_ Abbreviations: TC: total cholesterol, non-HDL-C: non-HDL cholesterol, LDL-C: LDL cholesterol (calculated), HDL-C: HDL cholesterol, TG: Triglycerides.

## FIGURE S1


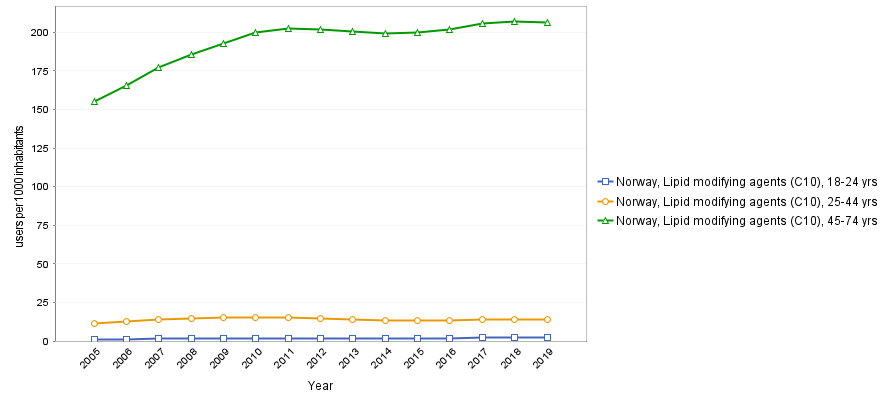


**FIGURE S1.** Users of lipid-lowering drugs per 1000 inhabitants in persons <45 years of age and ≥45 years of age in Norway, 2005-2019. Retrieved from the Norwegian Prescription Database, October 21th, 2021.

## Figure S2


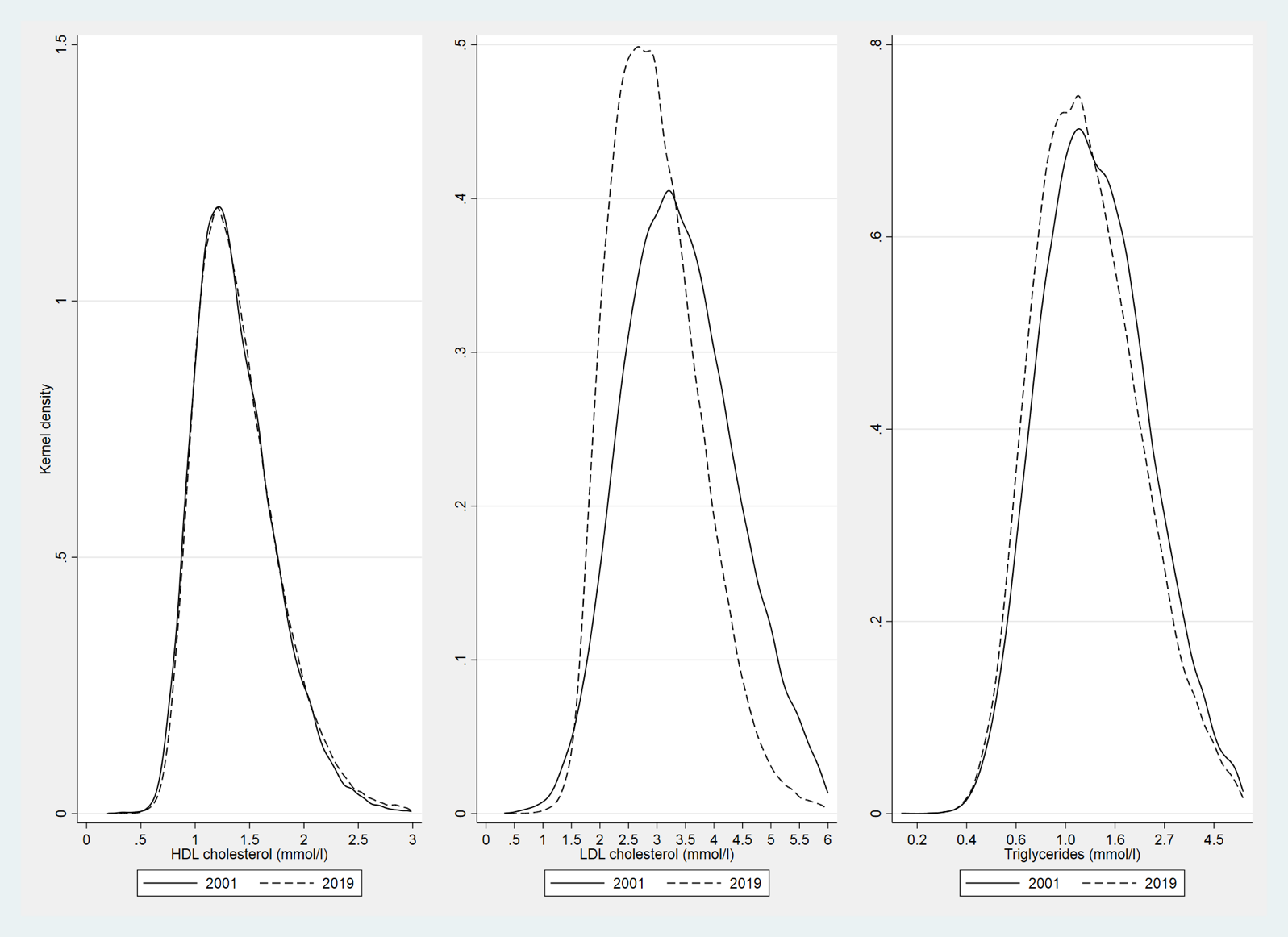


**FIGURE S2.** Population distributions of serum HDL cholesterol, LDL cholesterol and triglycerides in 18-49 year old men and women in 2001 (solid lines) and 2019 (dashed lines).

## Figure S3


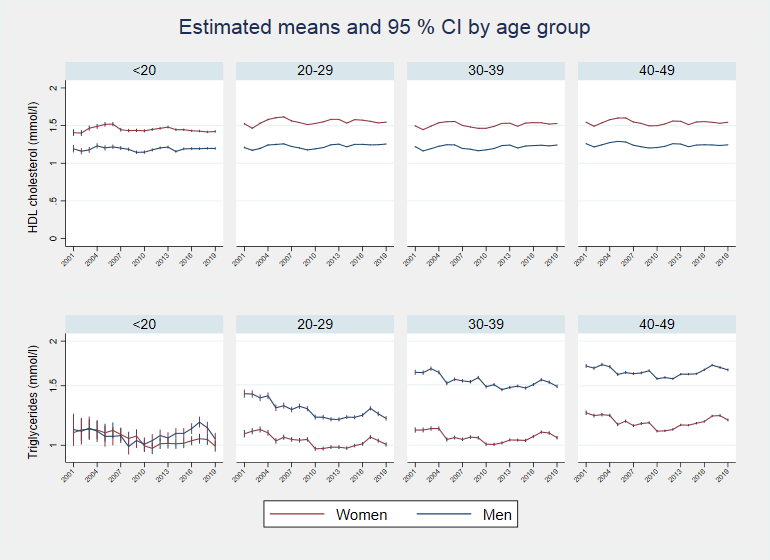


**FIGURE S3.** Trends in mean HDL cholesterol (top) and triglyceride (bottom) levels in 18-49 year olds by sex and age group, 2001 through 2019. 95 % confidence intervals are shown as spikes.

## Figure S4


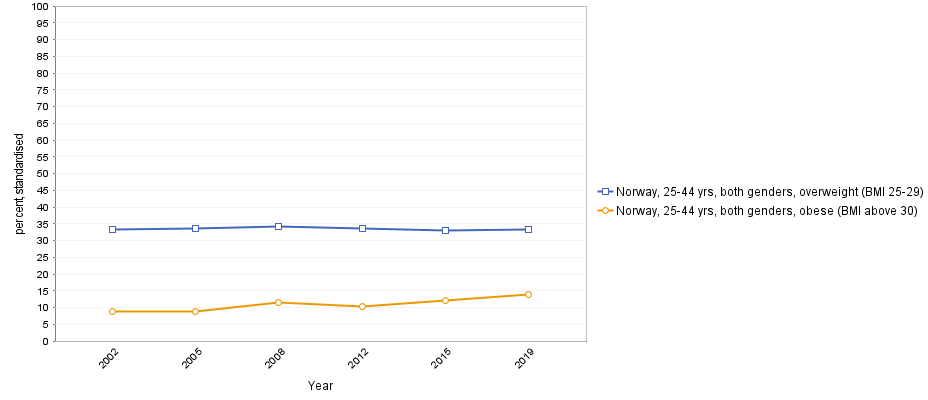


**Figure S4.** Prevalence of overweight and obesity in 25-44 year olds in Norway, 2002-2019. Retrieved from Norhealth (norgeshelsa.no), October 20^th^, 2021.
